# Flow synthesis of phenylserine using threonine aldolase immobilized on Eupergit support

**DOI:** 10.3762/bjoc.9.254

**Published:** 2013-10-22

**Authors:** Jagdish D Tibhe, Hui Fu, Timothy Noël, Qi Wang, Jan Meuldijk, Volker Hessel

**Affiliations:** 1 Micro Flow Chemistry and Process Technology, Department of Chemical Engineering and Chemistry, Eindhoven University of Technology, P.O. Box 513, 5600 MB Eindhoven, the Netherlands

**Keywords:** Eupergit, flow chemistry, immobilized enzyme, threonine aldolase

## Abstract

Threonine aldolase (TA) from *Thermotoga maritima* was immobilized on an Eupergit support by both a direct and an indirect method. The incubation time for the direct immobilization method was optimized for the highest amount of enzyme on the support. By introducing the immobilized TA in a packed-bed microreactor, a flow synthesis of phenylserine was developed, and the effects of temperature and residence time were studied in particular. Calculations of the Damköhler number revealed that no mass transfer limitations are given in the micro-interstices of the packed bed. The yield does not exceed 40% and can be rationalized by the natural equilibrium as well as product inhibition which was experimentally proven. The flow synthesis with the immobilized enzyme was compared with the corresponding transformation conducted with the free enzyme. The product yield was further improved by operating under slug flow conditions which is related to the very short residence time distribution. In all cases 20% diastereomeric excess (de) and 99% enantiomeric excess (ee) were observed. A continuous run of the reactant solution was carried out for 10 hours in order to check enzyme stability at higher temperature. Stable operation was achieved at 20 minute residence time. Finally, the productivity of the reactor was calculated, extrapolated to parallel run units, and compared with data collected previously.

## Introduction

Enzymes are bio-based catalysts having some distinct properties like high activity, high stereo-, regio- and chemoselectivity and high substrate specificity, which allow their use in a complex synthesis in a green and clean manner [1]. Recently, enzymes have received much attention for making processes more economically and ecologically beneficial, as they facilitate downstream processing requiring less separation steps, providing the best product quality with high purity at low energy consumption [2]. Still, the industrial applications of enzymes are hampered by many factors, like the lack of operational stability and the difficulty of enzyme recovery. Recently, Wang et al. have shown that the activity and recyclability of the CalB enzyme can be enhanced by the use of a polymersome Pickering emulsion. As such, a biphasic system could be used by loading the enzyme in the aqueous phase and organic reagents in the polymersome [3]. The above mentioned drawbacks may also be largely avoided by the use of an immobilized enzyme [4]. Immobilization has also been shown to enhance the stability of the enzyme [5]. A number of different immobilization methods were reviewed by Sheldon [6].

Despite the many advantages to use enzymes, their activity is often low, creating a demand for process intensification. In the last decade, different methods of process intensification have been proposed and tested for both fine-chemical and bulk-chemical processing [7–9]. It makes sense to test process intensification for biochemical processing as well. Microreactors, as a preferred process intensification tool, have gained considerable importance due to their many advantages over conventional batch reactors, including rapid heat and mass transfer, high surface area-to-volume ratios for dispersed media, and short processing times [10]. Indeed, there is indication that biocatalysis under flow conditions provided by a microreactor can result in better process control by external numbering-up in which each subunit of reaction can be examined separately with enhanced productivity [11].

Microreactors have been used in many fields of chemistry such as analytical systems [12], multiphase reaction systems [13–15], cross coupling reactions [16] and in chemical synthesis of pharmaceutical products [17]. Furthermore, the use of novel process windows to enhance the chemical production has also been reviewed [18–22]. From the industrial perspective, Hessel et al. have analyzed the patent situation in the field of microreaction technology [23]. In extension to such fine-chemical and pharmaceutical applications being investigated for almost 20 years, the quite recent introduction of microfluidic devices in bioprocess intensification and biocatalysis has been reviewed [24]. Asanomi et al. have also summarized the use of microfluidic devices in biocatalysis and compared them with conventional batch reactors [25]. The advantages of enzymatic microreactors have been demonstrated both in process development and for the production scale [26]. Similarly these reactors can provide high throughput opportunities, reduced reaction time with high conversion efficiency and high productivity per unit reaction volume for biocatalysis in fine chemistry [27]. These advantages have been demonstrated for reactions such as hydrolysis and esterification [27], oxidation and reduction [28], C–C bond formation [29], and polymerization [30].

Recent advancements in the field of enzymatic microreactors include the use of alginate/protamine/silica hybrid capsules with ultrathin membranes [31], monolithic enzyme microreactors [29,32], and biodegradable enzymatic microreactors based on surface-adhered physical hydrogels of PVA [33]. Babich et al. demonstrated the possibility of gram scale synthesis of phosphorylated compounds using phosphatase immobilized on Immobeads [34]. Buchegger et al. used a microfluidic mixer to study the pre-steady state development of an enzymatic bioreaction and found that the dynamics of a biochemical reaction can be studied in a few seconds [35].

Although many promising routes have been developed for the synthesis of chiral α-amino alcohols, these often depend on the use of toxic and expensive chiral ligands coupled to metal complexes [36]. Enzymes overcome these drawbacks as they are not toxic and they can be obtained easily from microorganisms. Threonine aldolases (TA) are a class of enzymes which is PLP (pyridoxal-5’-phosphate) dependent and can catalyze the aldol reaction between glycine and a variety of aromatic and aliphatic aldehydes [37–39]. The same enzyme can also catalyze the reverse reaction, i.e. the cleavage of threonine into glycine and acetaldehyde [40–42]. Fesko et al. conducted kinetic and thermodynamic studies using the phenylserine synthesis from glycine and benzaldehyde as a model reaction [43]. The same group also investigated the effect of ring-sided substituents of benzaldehyde on the product yield, revealing that TA accepts aromatic compounds having electron withdrawing groups as a substrate [44]. Aldolases are known to be hampered by thermodynamic and kinetic limitations, such as low diastereoselectivity and product yield. To overcome this drawback of aldolases, dynamic kinetic asymmetric transformation has been carried out in which a bi-enzymatic process was performed to achieve a high yield of the product by shifting the reaction equilibrium [45].

Eupergit oxirane acrylic beads provide a rapid and simple support for immobilization and have been used to immobilize various enzymes for a number of reactions [46]. Immobilization on Eupergit (a porous material) can be achieved without the need for any additional reagents, as the epoxy groups on Eupergit can react directly with the nucleophilic groups of the enzyme by forming strong covalent linkages like amino, hydroxy or mercapto functional groups. Eupergit has a high density of epoxy groups on the surface (oxirane density 300 μmol/g dry beads [47]), increasing the possibility of multipoint attachment of the enzyme. This multipoint attachment provides enhanced conformational stability, which translates to long term operational stability. Fu et al. have already investigated the thermal stability of TA on different supports [48] and found that the enzyme stability increases after immobilization on Eupergit support.

**Scheme 1:**
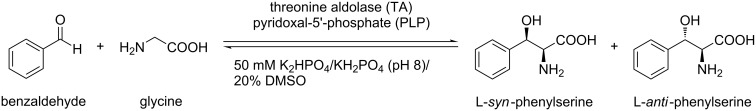
Phenylserine synthesis.

In continuation of our previous work [48], this paper includes further investigation of the thermal stability of TA on Eupergit by a so-called direct and indirect method. Also, a flow synthesis of Eupergit-immobilized TA in a packed bed microreactor was established and compared to the use of free enzymes in the reaction of glycine and benzaldehyde. The reaction investigated is the synthesis of phenylserine starting from benzaldehyde and glycine (Scheme 1). The segmented flow experiments were carried out as an alternative to the free enzyme in a single phase flow to maximize the yield. A continuous activity check was performed for determining the stability of the immobilized enzyme over a longer period of time. The productivity of the flow systems reported here was also determined and compared with the performance published in previous literature.

## Results and Discussion

### Comparison of direct and indirect enzyme immobilization

For the direct immobilization method, 99% enzyme retention and 52% activity retention were observed, while in the indirect method the values were 78% and 89%, respectively (Table 1). In case of direct immobilization, the enzyme and support are both directly in contact with each other which resulted in higher values for enzyme retention. For the indirect method, however, the epoxy groups were converted into aldehyde groups. These groups can only react with nucleophiles such as amino groups. During treatment of Eupergit with ethylenediamine, a coupling of two adjacent epoxy groups on the support can occur which would result in a lower amount of enzyme retention. The multipoint attachment in case of direct immobilization may block the active site of the enzyme and, as a consequence, this would result in a lower degree of activity retention. In case of the indirect method the enzyme was separated from the support by a spacer element. This was formed by the reaction of ethylenediamine and glutaraldehyde. This reaction reduces the probability of blocking the active site of the enzyme which would provide higher activity retention. For the direct method, our results demonstrate a high activity to those reported in the literature. There, only 52.4% enzyme retention and activity retention of 20.7% were achieved while for the indirect method 37% enzyme loading with 31% of activity retention was observed [49].

**Table 1:** Comparison of direct and indirect method.

Method	Enzyme retention (%)	Activity retention (%)

Direct	99	52
Indirect	78	89

### Comparison of thermal stability of TA immobilized by two different methods

**Figure 1:**
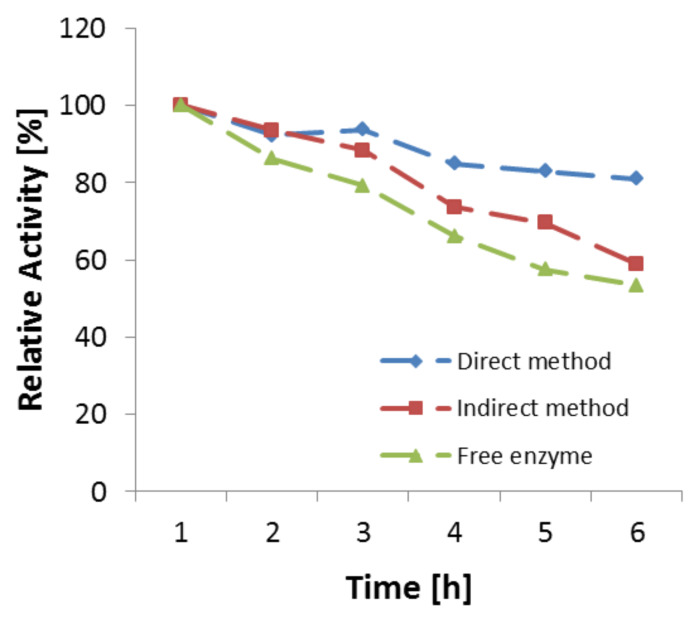
Activity loss of TA immobilized by two different methods and as a free enzyme at 80 °C. Reproduced with permission from Elsevier [48].

For TA immobilized by using the direct method, the increase in stability of the enzyme is likely due to a multipoint attachment. Around 80% of activity retention was observed after 5 hours. The multipoint attachment may provide a rigid structure for the enzyme and increased stability. In case of the indirect method the enzyme almost behaves like a free enzyme, which is due to the introduction of spacer between the enzyme and the support. This enhances the conformational freedom of the enzyme. The behavior of the curve for the three cases free, indirect immobilized and direct immobilized TA is shown in Figure 1.

### Optimization of immobilization time

**Figure 2:**
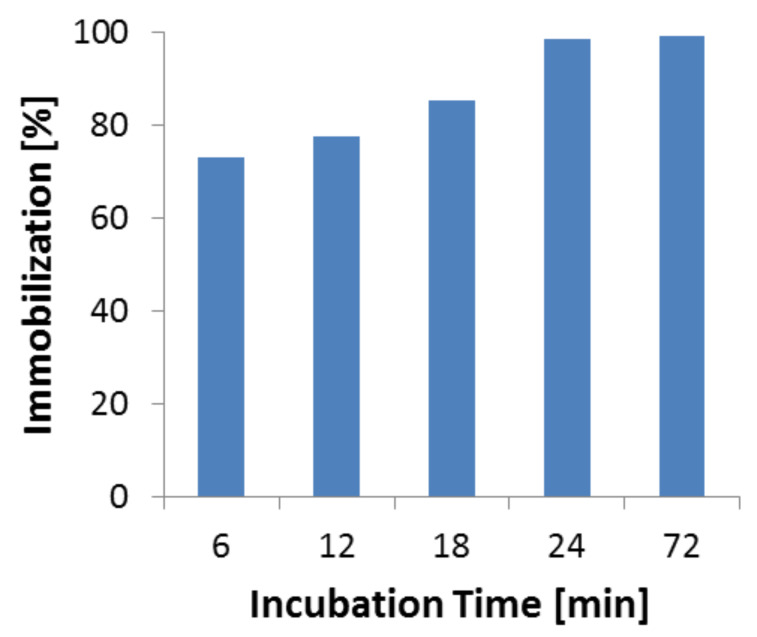
Degree of immobilization versus incubation time.

The optimization of immobilization is discussed here only for the direct method. In order to make the process more feasible and cost effective for industrial applications, we reduced the time of immobilization of TA on Eupergit [50]. The amount of immobilized enzyme increases until it reaches a plateau after about 24 hours (Figure 2). At this point 99% of enzyme immobilization was achieved which is a substantial improvement with respect to 72 hours originally employed.

### The phenylserine synthesis in batch using free enzyme

**Figure 3:**
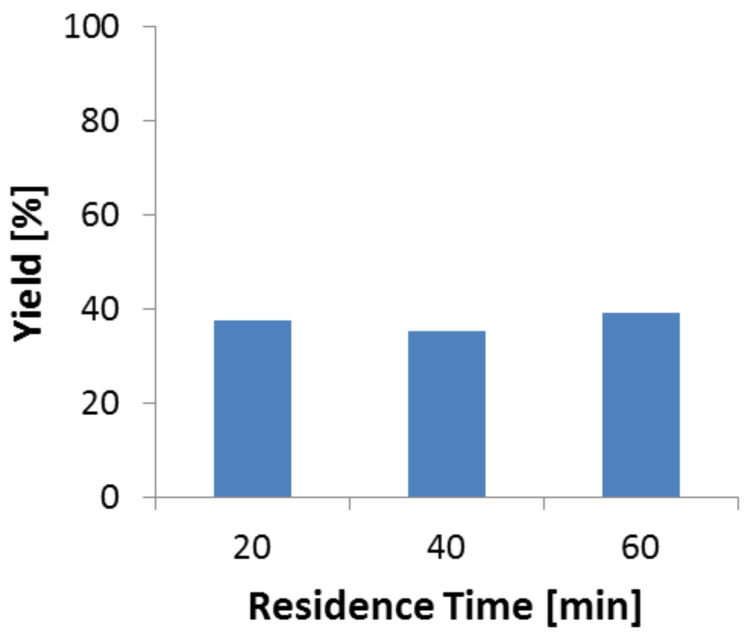
Batch reaction using free enzyme. Reaction conditions: Reaction volume (10 mL), TA (2.7 mg, specific activity – 0.135 U/mg), temperature (70 °C).

Batch reactions were carried out to estimate the yield of phenylserine formation and reaction time which can principally be achieved. Flow processing, and in particular its productivity highly depends on achieving a complete transformation in a very short time, ideally within a few minutes or less. After 20 minutes, a 40% yield of phenylserine was achieved and no further yield increase was observed (Figure 3). TA-based reactions are known to be of equilibrium-type which limits the achievable yield to about 40% in the given case. Another reason could principally be deactivation of the enzyme. Yet, this is not so likely, since Figure 1 shows that even after 5 hours about 50% of the enzyme still shows activity. Thus, a self-inhibitory effect of the product on the TA at a given concentration can be made responsible or alternatively the reactant glycine which is present in high concentration could cause inhibition [51].

### Product inhibition study

**Figure 4:**
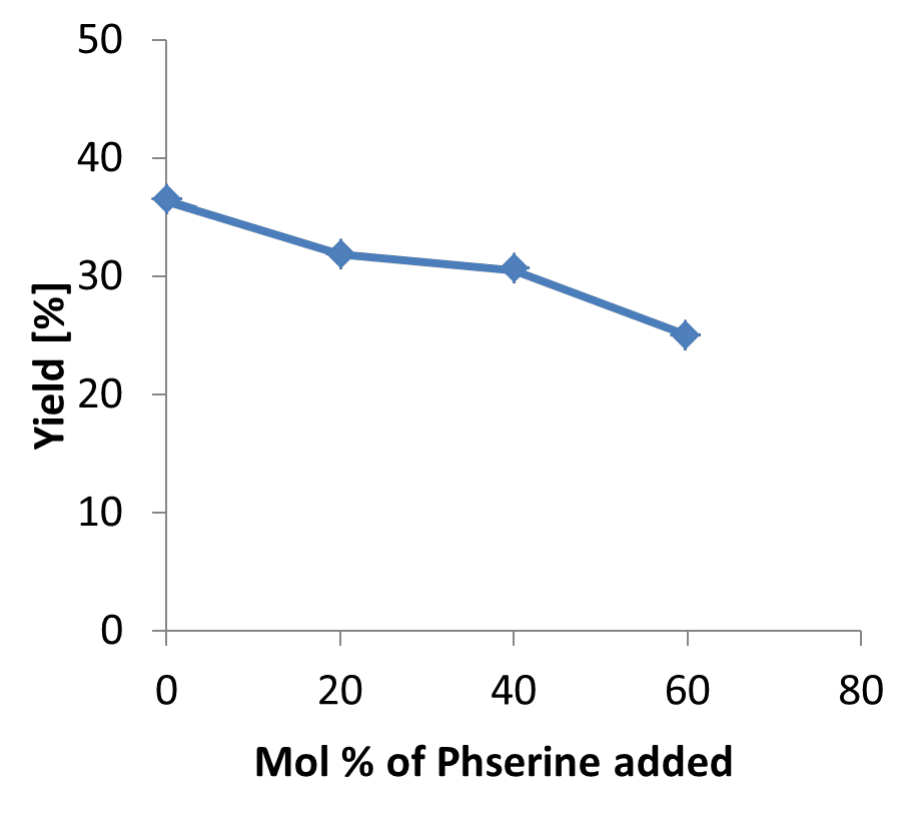
Product inhibition study.

The product inhibition study was carried out in order to understand whether there is an effect of product formed during the reaction which might block the active site of the enzyme. To achieve this we carried out three reactions for 40 minutes at 70 °C. The amount of product which was added before the reaction is 20, 40 and 60 mol % (Figure 4). It has been observed that when the amount of product increases the yield starts to decrease which indicates that the product inhibition effect does exist. This is due to the very high concentration of product as compared to the amount of enzyme used for the reaction.

### Synthesis of phenylserine by using immobilized TA in a microreactor

#### Effect of temperature

**Figure 5:**
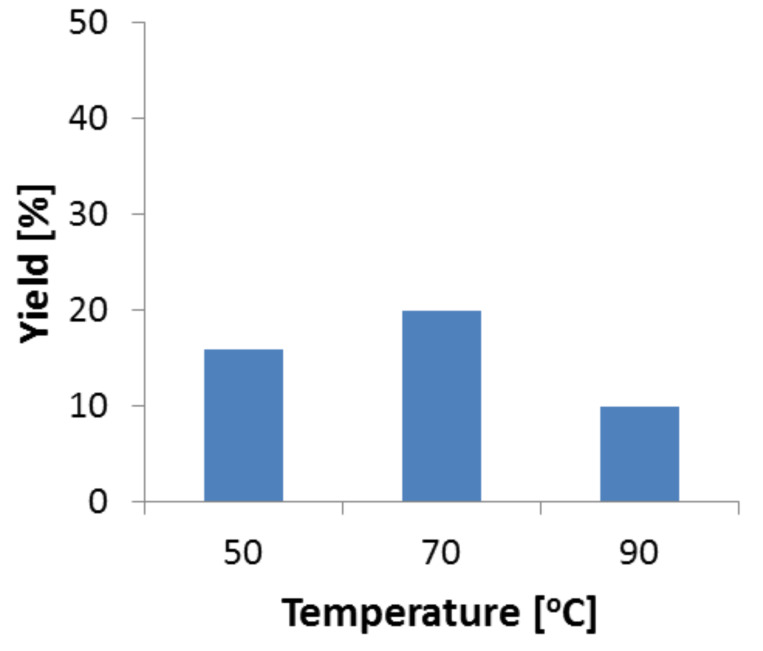
Effect of temperature on product yield. Reaction conditions: Reaction volume (0.250 mL), TA (1.1 mg, specific activity – 0.052 U/mg), flow rate (25 μL/min). Samples were taken after 30 minutes at the indicated temperature.

The effect of the reaction temperature on the yield of phenylserine was investigated at a residence time of 10 minutes (flow rate = 25 μL/min). As shown in Figure 5, the yield increased up to a temperature of 70 °C reaching a value of maximal 25% which is in line with the limitations based on the equilibrium discussed before. Above 70 °C, a decrease in yield was observed (Figure 5). It can be assumed that above 70 °C the reaction rate is even higher, however enzyme deactivation becomes more substantial. Therefore, we chose 70 °C as the optimum temperature for the following experiments.

#### Effect of flow rates

**Figure 6:**
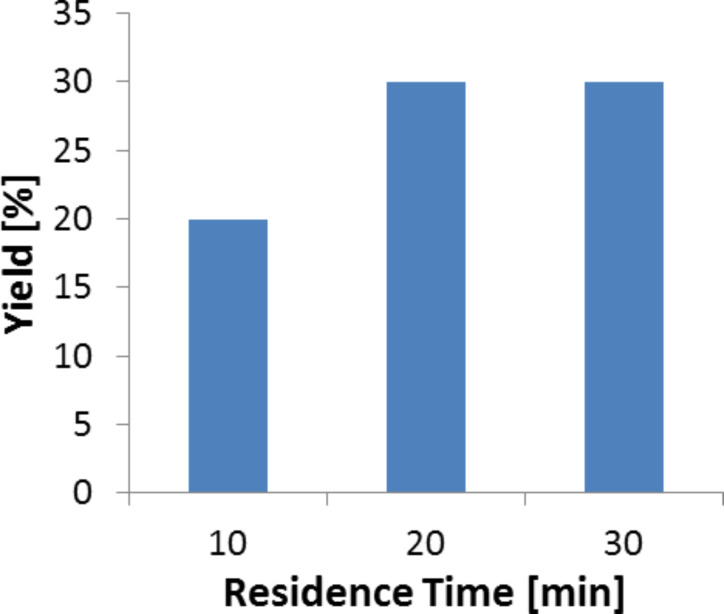
Effect of flow rates on yield for immobilized enzymes in a packed bed microreactor (70 °C). Reaction conditions: Reaction volume (0.250 mL), TA (1.1 mg, specific activity – 0.052 U/mg), temperature (70 °C).

When operating the packed bed microreactor with immobilized TA at 70 °C, the yield of phenylserine can be further increased to 30% at longer residence times (Figure 6). This result resembles the batch performance reported above. The reason can be the already supposed product inhibition of the enzyme or a specific flow effect. At higher flow rates and shorter residence times the product yield decreases. In order to check whether mass transfer limitations are involved, we estimated the mass transfer coefficient for transport of the substrate to the particles.

#### Mass transfer calculations

The calculation of the Damköhler number was carried out for the reaction with immobilized TA under the same conditions discussed before in the section above*.* The following values were used to calculate the mass transfer coefficient, porosity (ε) = 0.5 estimated, density (ρ) = 1024 kg/m^3^, viscosity of fluid (μ) = 0.00134 kg/ms and thermal conductivity (*K*) = 0.58 (W/m·K).

The Reynolds number (Re) for a fluid flow through a bed of approximately spherical particles of diameter *D* can be calculated, according to Equation 1 in which the void fraction is ε and the superficial velocity is *V*.

[1]
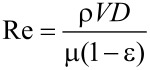


The Reynolds number was 0.1 and the Schmidt (Sc) number was calculated to be 1.309. Next, the Sherwood number (Sh) was calculated using the following equation,

[2]
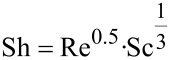


The Sherwood number was found to be 1.093 which provided a mass transfer coefficient (*k*) of 17 × 10^−6^. The reaction rate *v*_max_ was calculated using experimental data and the value was 1.67 × 10^−6^ mol/m^2^s. This allowed us to determine the Damköhler number (Da) using following equation,

[3]
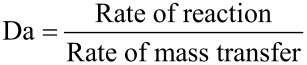


The Damköhler number turned out to be 0.98 × 10^−3^, which is much smaller than the threshold criterion 1. Therefore, no mass transfer limitation can be encountered in the present case.

#### Free enzymes in flow using a capillary microreactor

Some flow experiments were performed in Teflon tubing at 70 °C with the free enzyme and unraveled an almost linear increase of product yield with increasing residence times. Under these homogeneous conditions, very short diffusion distance between the reactants and the enzyme is guaranteed. Yet, the yield does not increase above 15% for a residence time of 20 minutes which is about half compared to heterogeneous conditions. This could point towards a higher enzyme activity and/or to higher enzyme loadings in the immobilized state.

**Figure 7:**
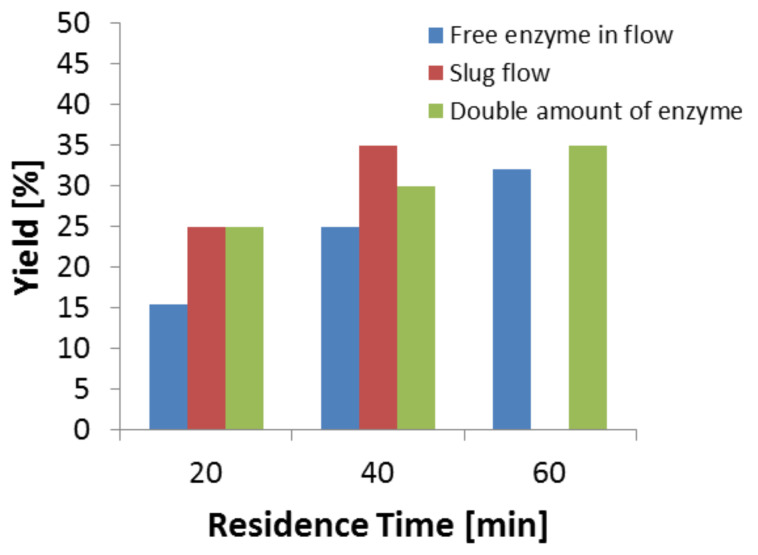
Effect of residence time on yield for free enzymes in a Teflon tube microreactor at 70 °C. Reaction conditions: For free enzyme in flow: Reaction volume (0.500 mL), TA (0.135 mg, specific activity – 0.135 U/mg). Slug flow: Reaction volume (0.500 mL), TA (0.135 mg, specific activity – 0.135 U/mg). Double amount single phase: Reaction volume (0.500 mL), TA (0.27 mg, specific activity – 0.135 U/mg).

For a residence time of 60 min (Figure 7) a yield of 32% was achieved, which is not substantially higher than found for the best yield using the immobilized enzyme under microreactor operation conditions. Studies under even longer residence times could not be conducted, since the corresponding flow rates could not be accommodated with the pumps used.

Next, segmented flow experiments were carried out by injecting argon in the tube microreactor which generated a slug flow with a flow rate of 12.5 µL/min and 6 µL/min, respectively, which corresponded to residence times of 20 and 40 minutes. We aimed to achieve a much narrower residence time distribution under slug flow conditions as opposed to the laminar parabolic flow profile of the single-phase flow system. The latter can lead to a considerable share of reactants experiencing a (much) shorter residence time than the given (averaged) residence time based on the nominal flow rates. Under slug flow conditions, all reactants will see almost the same residence time due to reduced axial dispersion allowing the reactor to operate as an ideal plug flow reactor. Additionally, slug flow is known to create profound transversal recirculation patterns within the liquid slugs which constitute a permanent highly mixed fluid system. Single-phase flow operations do not show similarly strong passive mixing. However, as no mass transfer limitations exist for the immobilized enzyme which also should apply here, the mixing issue can be regarded of less relevance than the residence time control issue.

Indeed, the slug flow derived yields are higher compared to the single-phase flow process. The product yield for the slug flow process with a residence time of 20 min, is lower compared to the flow operation with the immobilized enzyme. For a residence time of 40 min the product yield is 34%. Thus, it is slightly higher than the best yield obtained for a flow reactor using the immobilized enzyme as packed bed. When comparing this result with the free enzyme reaction using a capillary microreactor, the yield increases from 16 to 25% (20 min residence time) and only a 5% increase of the yield for 40 min residence time. Obviously, slug flow operation does have a slight effect on yields. When the amount of enzyme was doubled for a single-phase flow experiment using a Teflon tube microreactor, the product yield improved from 16% to 25% (20 min residence time). A longer residence time of 40 min further increases the yield to 30% which did not change when employing a residence time of 60 min. Again, either deactivation of TA or product inhibition can be made responsible as discussed above.

#### Long term enzyme stability

**Figure 8:**
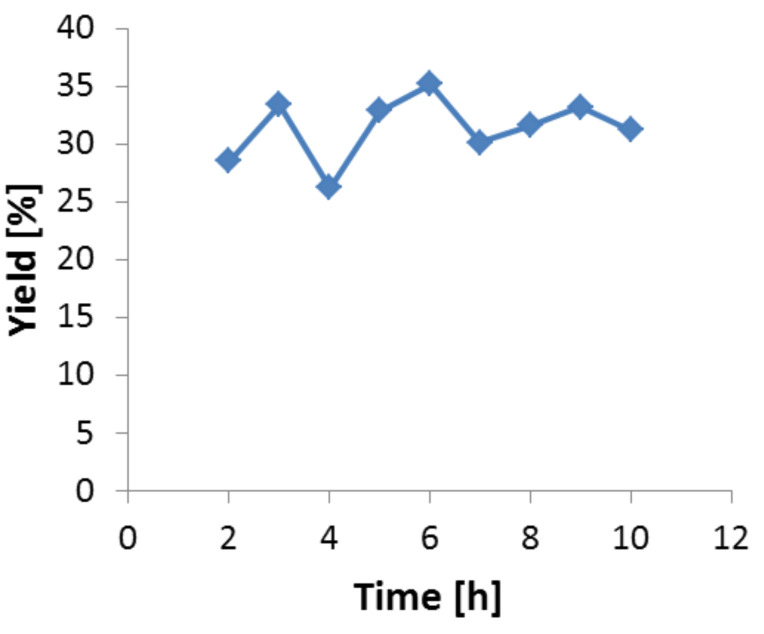
Long term enzyme stability at 70 °C. Reaction conditions: Reaction volume (0.250 mL), TA (1.1 mg, specific activity – 0.052 U/mg), flow rate (12.5 μL/min), temperature (70 °C).

A continuous experiment was performed to study the long term stability of immobilized enzyme at 70 °C. The stability of Eupergit-bound TA (direct method) has already been discussed. The studies revealed that at 80 °C there is 20% loss of enzyme activity within 4 hours. It was anticipated that at lower temperatures the lifetime of the immobilized enzyme would be extended and would allow long term operation of the flow system. Indeed, the product yield was almost constant at around 30–35% even after 10 hours of continuous operation (Figure 8). Here, the inhibitory effect by the product might be reduced due to the large amount of enzyme (1.1 mg) and the small reactor volume of about 250 µL. The continuous removal of the product from the reaction system may reduce this effect. This signifies the stable operation of the immobilized enzyme over a longer period of time which is useful to reduce the cost of the process by reusing the catalyst.

## Conclusion

**Scheme 2:**
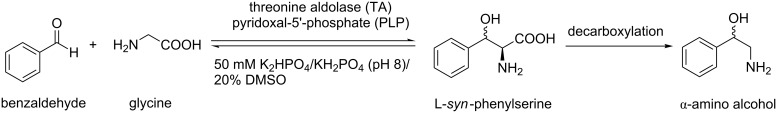
Synthesis of chiral α-aminoalcohol by telescoping aldolase reaction with decarboxylation.

To the best of our knowledge, we report the first use of immobilized threonine aldolase in a microreactor for the flow synthesis of phenylserine. So far, we achieved a maximum of about 30% yield under the typical flow rates using TA bound to Eupergit. The yield was about 35% for the free enzyme using a tubular microreactor. Here slug flow performed slightly better than single-phase flow operation. Several explanations have been provided which rationalizes the findings reported above. These include the residence time distribution present for the single-phase flow operation, different degrees of enzyme deactivation, and different degrees of enzyme activity – each being different for the three flow operations investigated. This gives room for future optimization. Above all, however, the existence of an equilibrium between starting materials and products is the major factor of being restricted to a maximum of 40% yield. Here, removal of the product from the enzyme thereby readjusting the equilibrium is the method of choice. One opportunity to achieve this could be the decarboxylation of phenylserine as depicted in Scheme 2 which is currently been investigated in our laboratories.

Taking into account the experimentally derived flow performance mentioned above, the calculated TA-enzyme based microreactor productivity was found to be 5.4 mg/h. From an industrial perspective on microreactors, it is reasonable to assume that an external numbering-up of 10 (respectively, 54 mg/h) along with scaling-out of those reactors the production of 200 mg/h in total can be achieved. This value is not too far away from the production need for commercial high-value products in pharmacy at a price of 500 €/g and higher. The comparison between theoretical and experimental values for productivity has been shown in Table 2.

**Table 2:** Comparison of productivity between the theoretical calculations and experimental observations.

Calculations	Specific activity of enzyme (U/mg)	Amount of Eupergit inside reactor (mg)	Productivity values (g/h)

Theoretical^a^	0.188	133	0.280
Experimental	0.135	125	0.0054

^a^see [48].

Both the theoretical and experimental productivities are based on a quite low (experimentally used) amount of active enzymes; simply for reasons of availability. In [48] we calculated also productivities of other enzymatic microreactors (with other enzymes, having no availability limitation) and this result in much higher productivities.

In this paper, we made a first step towards a critical view on the commercial potential for high-priced pharmaceutical products using enzymatic microreactor technology.

## Experimental

### Materials

The enzyme L-*allo*-threonine aldolase (L-low-TA) (EC 4.1.2.48) having concentration of 3 mg/mL and activity of 0.135 U/mg has a strong preference for L-*allo*-threonine from *Thermotoga maritima* and was kindly donated by the Junior Research Group ‘‘Industrial Biotechnology’’ (University of Leipzig, Germany). L-threonine, β-nicotinamide adenine dinucleotide disodium salt (NADH) were purchased from AppliChem GmbH (Darmstadt, Germany). Glycine, benzaldehyde, pyridoxal-5’-phosphate (PLP), Eupergit CM, 2-mercaptoethanol, 25% glutaraldehyde solution in water, ethylenediamine and other reagents were all purchased from Sigma Aldrich (Zwijndrecht, NL) and used as received. For immobilization of TA on Eupergit 1 M phosphate buffer solution was used, while for the phenylserine synthesis a 50 mM solution was used.

### TA immobilization

**Figure 9:**
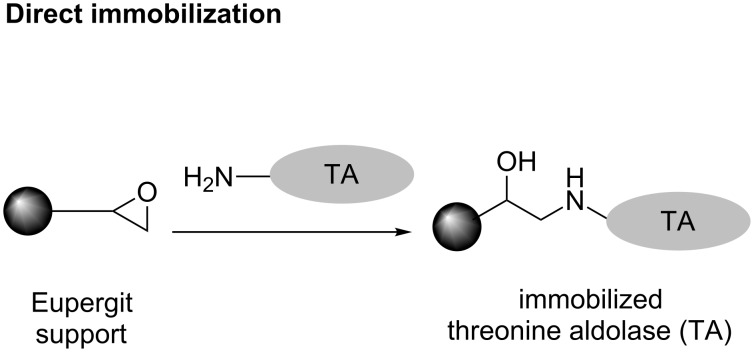
Direct immobilization.

**Figure 10:**
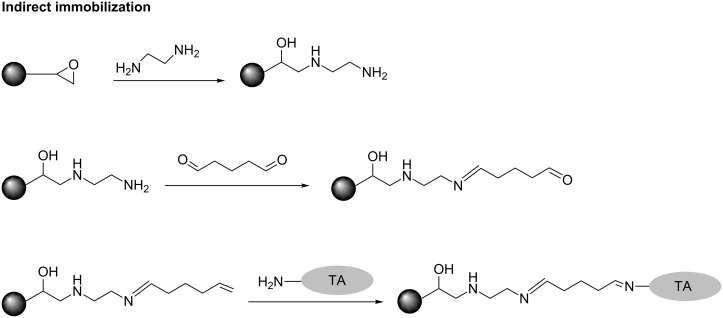
Indirect immobilization.

The immobilization was carried out by a direct and an indirect method. For the direct method, the enzyme was bonded directly to the surface of the support, while the indirect method a spacer is used to provide enhanced enzyme mobility. The experimental procedure for direct immobilization (Figure 9) and indirect immobilization (Figure 10) as well as enzyme retention, activity retention and determination of enzyme concentration are well explained in [48].

### Immobilization at different incubation times

At first the immobilization was carried out using the procedure given in literature [51]. 500 mg of Eupergit was treated with 2.5 mL of the TA enzyme solution (4.365 mg of TA) in a test tube at room temperature. The test tube was then kept in an orbital shaker for a predetermined time. Five different contact times were determined (6, 12, 18, 24 and 72 h).

### Enzyme reactions

#### Batch reactions

Batch reactions were carried out in 20 mL test tubes at 70 °C, while stirring. The test tube was charged with 750 mg glycine (1 M), 106 mg benzaldehyde (0.1 M), 100 μL 5 mM PLP solution, 2 mL DMSO, 0.9 mL (2.7 mg) TA solution (activity = 0.407 U/mL) and 7 mL of a 50 mM phosphate buffer solution. The samples were collected at three different reaction times (20, 40 and 60 min). In each case 1 mL of sample was collected and the reaction was terminated by adding a 30% trichloroacetic acid solution. Then, all samples were extracted with 2 mL of internal standard solution (1,3-dimethoxybenzene in ethyl acetate). The enantiomeric excess (ee) and the diasteriomeric excess (de) of phenylserine were determined by HPLC analysis of the aqueous phase, while conversion of benzaldehyde was determined from analysis of the organic layer using gas chromatography. Since no byproducts could be detected, the degree of conversion reflected the yield of phenylserine formation.

#### Flow reactions

**Figure 11:**
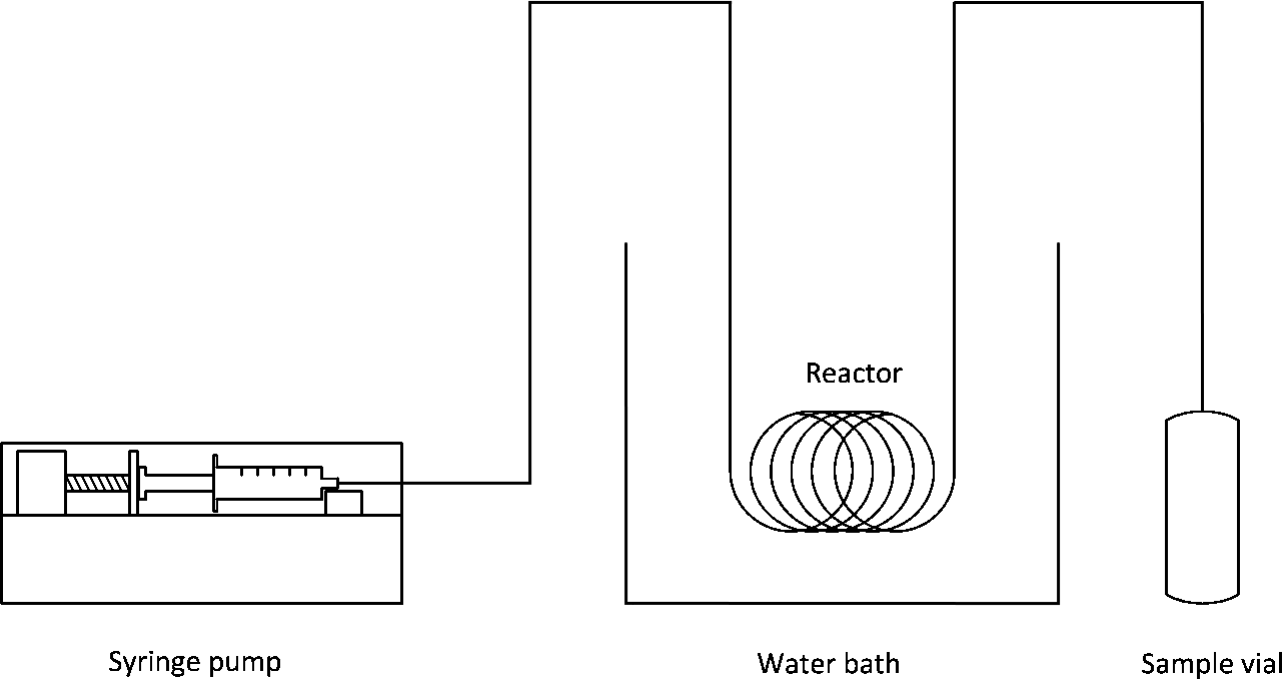
Flow reaction set-up using free enzyme.

A Teflon tubing with an inner diameter of 500 μm and a volume of 500 μL was used to perform the flow experiments. The tubing was completely immersed into the thermostat bath at 70 °C. Amounts of reactants employed were kept similar as for the batch experiments. The solution was taken up in a single syringe and pumped at different flow rates (25 μL/min, 12.5 μL/min, 6 μL/min). The samples were collected at the outlet and immediately hydrolyzed by adding a 30% solution of trichloroacetic acid. For each reaction three samples were collected. Then a sample was taken from the syringe after 12 hours and conversion was determined to be 7%. The setup for the reaction is shown in Figure 11.

#### Immobilized enzyme reaction using micro flow

**Figure 12:**
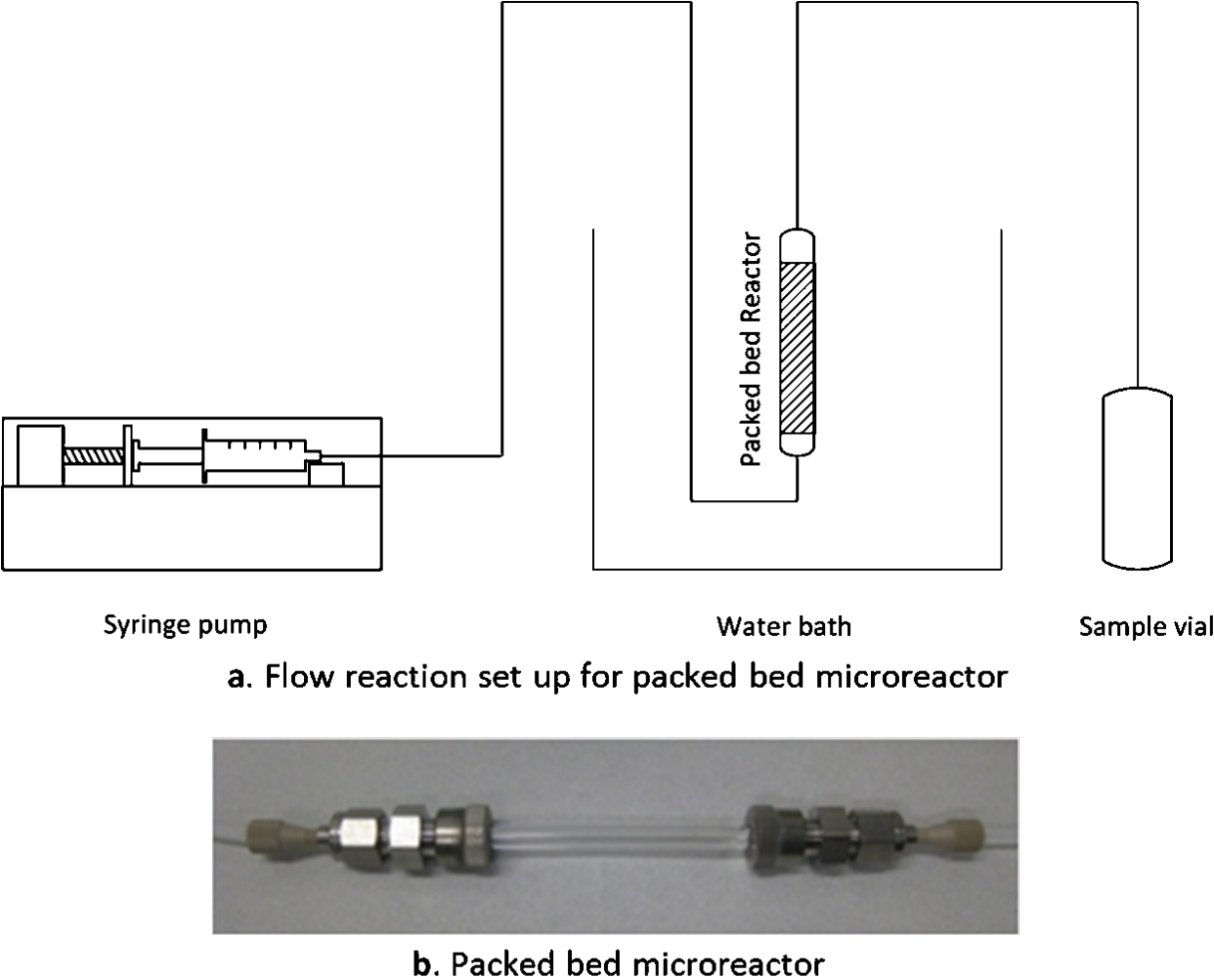
Experimental setup for packed be microreactor.

A simple glass tube with an inner diameter of 3.5 mm and a length of 5 cm was used as housing for the TA immobilized Eupergit support. A neck was constructed at the outlet for holding the glass wool which prevented wash out of the immobilized support from the reactor. In each experiment the same amount of Eupergit (125 mg corresponding to dry wet of Eupergit) was used as fixed-bed material. The length of the Eupergit bed was determined to be 4.7 cm +/− 0.2 cm, while the volume of the filled reactor was around 250 μL. The reactor was kept in a vertical position during all experiments (Figure 12a and b). During operation the reactor was incased in a thermostat. The reaction mixture contained 750 mg (1 M) of glycine, 106 mg (0.1 M) of benzaldehyde, 100 μL of 5 mM PLP solution, 2 mL of DMSO, and 7.9 mL of 50 mM phosphate buffer solution. For each experiment a fresh batch of immobilized TA was used. For each reaction three samples were collected and the average was calculated.

### Analysis of the reaction

#### Determination of conversion (yield)

The conversion of benzaldehyde was determined by GC–FID (Varian 430-GC) having a CP-Sil 5 CB column of 30 meters (0.25 mm ID with film thickness of 1 micron), and a flame ionization detector. The carrier gas was helium (1 mL/min, split ratio 100). The oven temperature was maintained at 100 °C for 1 minute and then increased to 250 °C at a rate of 25 °C/min. The injector temperature was fixed at 250 °C and the detector temperature at 250 °C.

#### Determination of enantiomeric excess (ee) and diastereomeric excess (de)

**Figure 13:**
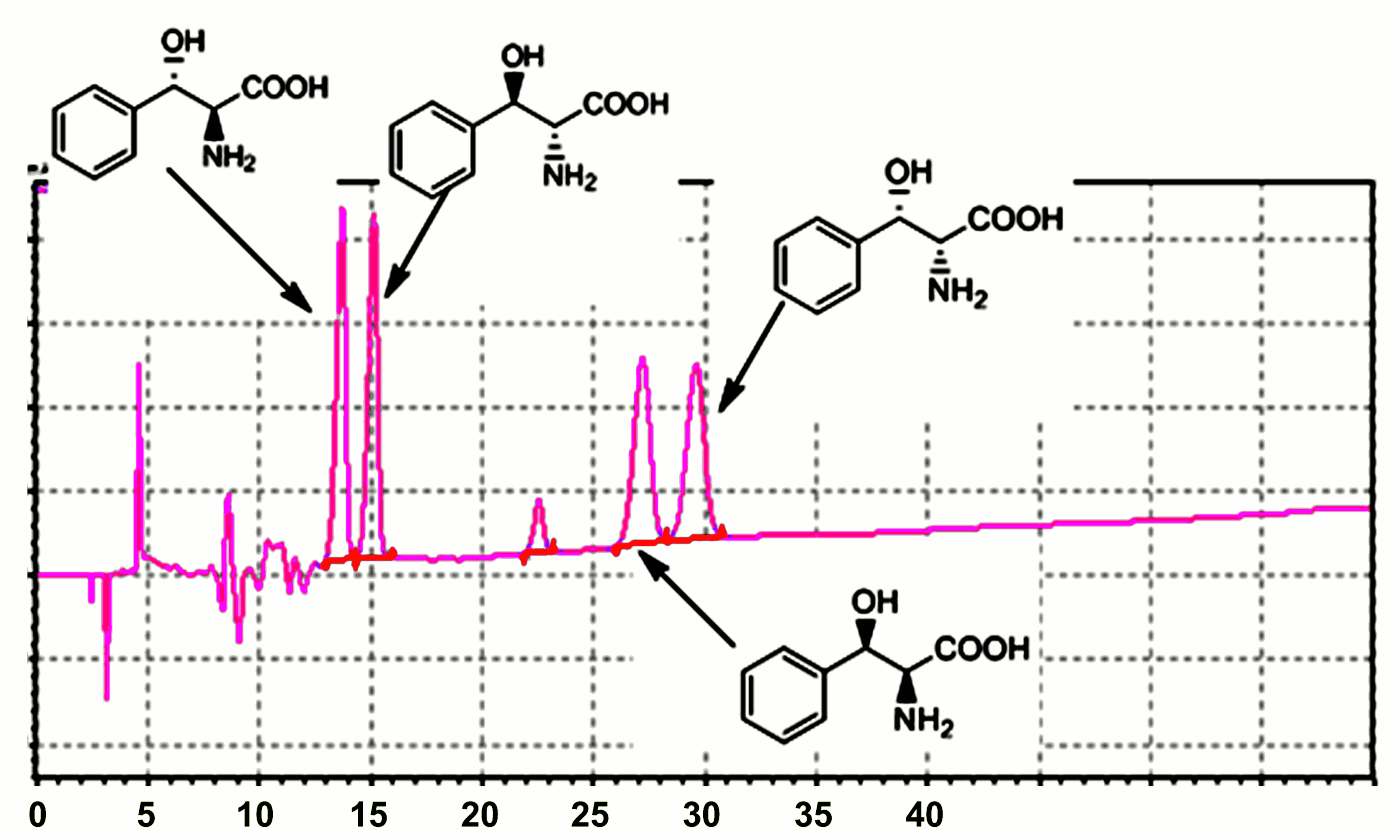
Analysis of the four isomers of phenylserine on a chiral column.

Determination of the enantiomeric excess (ee) and the diastereomeric excess (de) was achieved by HPLC using a chiral column (Chirex 3126 (D)-penicillamine Column 250 × 4.6 mm) under the following conditions: 75% 2 mM CuSO_4_ solution + 25% methanol at a flow rate of 1 mL/min. Detection of products was achieved at a wavelength of 254 nm. The column temperature was kept at 40 °C throughout the analysis. The separation of all the four isomers is depicted in Figure 13. The correct elution of the different stereoisomers was elucidated by injecting pure sample of each compound and comparison with literature [43–45].

In all experiments, 20% of diastereomeric excess of phenylserine was observed and a >99% enantiomeric excess was obtained for both diastereomers.
